# Monitoring and Optimisation of Ag Nanoparticle Spray-Coating on Textiles

**DOI:** 10.3390/nano11123165

**Published:** 2021-11-23

**Authors:** Sara Trabucco, Simona Ortelli, Benedetta Del Secco, Ilaria Zanoni, Franco Belosi, Fabrizio Ravegnani, Alessia Nicosia, Magda Blosi, Anna Luisa Costa

**Affiliations:** 1 ISAC-CNR, Institute of Atmospheric Sciences and Climate-National Research Council of Italy, Via Gobetti 101, 40129 Bologna, Italy; s.trabucco@isac.cnr.it (S.T.); b.delsecco@isac.cnr.it (B.D.S.); f.belosi@isac.cnr.it (F.B.); f.ravegnani@isac.cnr.it (F.R.); a.nicosia@isac.cnr.it (A.N.); 2 ISTEC-CNR, Institute of Science and Technology for Ceramics-National Research Council of Italy, Via Granarolo 64, 48018 Faenza (RA), Italy; ilaria.zanoni@istec.cnr.it (I.Z.); magda.blosi@istec.cnr.it (M.B.); anna.costa@istec.cnr.it (A.L.C.)

**Keywords:** Ag nanoparticles, spray-coating, monitoring measurements, process optimization, particle release

## Abstract

An automatic lab-scaled spray-coating machine was used to deposit Ag nanoparticles (AgNPs) on textile to create antibacterial fabric. The spray process was monitored for the dual purpose of (1) optimizing the process by maximizing silver deposition and minimizing fluid waste, thereby reducing suspension consumption and (2) assessing AgNPs release. Monitoring measurements were carried out at two locations: inside and outside the spray chamber (far field). We calculated the deposition efficiency (E), finding it to be enhanced by increasing the spray pressure from 1 to 1.5 bar, but to be lowered when the number of operating sprays was increased, demonstrating the multiple spray system to be less efficient than a single spray. Far-field AgNPs emission showed a particle concentration increase of less than 10% as compared to the background level. This finding suggests that under our experimental conditions, our spray-coating process is not a critical source of worker exposure.

## 1. Introduction

Spray-coating techniques are widely used in a range of industrial fields, from graphic art applications to coatings and painting. Providing excellent coating on a variety of different shaped surfaces, these techniques are frequently part of in-line production systems. Advantages include minimum fluid waste, easy film thickness and roughness control, and the use of a broad spectrum of different viscosity fluids for a wide range of different coating requirements. In-depth study of the deposition technique can lead to process optimization, i.e., maximizing the material deposited and minimizing dispersal. Furthermore, the technique adopted in this study has considerable scalable promise. The simple operational process and design of the computer-controlled spray coaters would facilitate the transition to a full-fledged industrial process [1].

Monitoring spray-coating methods involves analyzing the different process parameters: spray rate, atomization air pressure, inlet- and exhaust air temperature, inlet- and exhaust air flow, nozzle size, and nozzle-to-bed distance [2]. The spray droplet size is a critical factor, determining the coating quality but also related to the spray rate and pressure. In general, the droplet size increases with increasing spray rate but decreases on increasing the spray pressure. The spray rate must not only be set to ensure the optimal spray droplet size but must be calibrated to prevent sticking. The coating quality is also strongly dependent on the method and equipment used during the application process.

The conventional method of monitoring the coating process has been the assessment of the weight of the support to be coated before and after the process. However, weight-checking support involves interrupting the coating process before completion of the cycle. Recent process analytics now allow real-time non-invasive quantitative monitoring of on- and in-line coating applications [3,4,5,6,7].

The atomized droplets sprayed during coating processes today often contain suspensions of various nanoparticles (NPs), which will form the surface coating once the liquid solvent has evaporated. This raises the important issue of NP aerosolization and consequent potential inhalation exposure. In fact, spray-coating processes are among the most critical in terms of occupational exposure, with industrial spraying processes among the primary causes of fine and ultrafine particle (UFP) dispersion at coating sites [8]. The inhalation exposure route has been identified as the most likely during processes involving aerosol dispersion. In addition, inhalation is generally considered the primary exposure route since the matrix includes small droplets of aerosolized UFP and nanoforms (NFs) that can easily reach the lung tissue [9]. The inhalation exposure is strongly influenced by process parameters such as the spray pressure, which conditions both the particle aerosolization and size distribution of the released particles [10].

We used a spray process to deposit a silver nanoparticles (AgNPs)-based coating on polyester fabric substrates to obtain antibacterial textiles. Textiles are suitable substrates for micro-organisms growth, especially at humidity and temperature conditions found in contact with the human body. The recent outbreak of the COVID-19 virus and recent increasing public concern about hygiene have urged the need for the development of textiles with antimicrobial/antiviral properties. A huge market for antibacterial textiles is foreseen for public facilities such as hospitals, health-care residences, hotels, airplanes, trains, etc., since they are an integral part of modern life. The application of inorganic NPs, including silver, is seen as a promising option to develop of durable (long lasting) antibacterial textiles [11,12,13]. The use of AgNPs incorporated in medical textiles is among the most promising strategies not only to provide the aseptic conditions in hospitals but also to overcome the emerging COVID-19 pandemic [14]. Specifically, a patented sol–gel synthesis of hydroxyethylcellulose (HEC) capped silver nanoparticles (Ag-HEC) [15] was used as the spray feed in a lab-scaled automatic spray-coating machine. The spray process was investigated with the dual purpose of (1) optimizing the process, maximizing the amount of silver deposited as a coating, reducing the suspension consumption, minimizing fluid waste and (2) assessing AgNP release inside and outside the deposition chamber. Both aspects are part of a safe and sustainable-by-design approach that aims to improve the antimicrobial textile production process by finding the optimal balance between input (feed) and output (waste) materials, and controlling and minimizing the emission determinant. Measurements were carried out using instrumentation able to provide real-time data and perform sampling for off-line characterization.

## 2. Materials and Methods

### 2.1. Materials and Process Description

Monitoring measurements were performed during a spray-coating process with an aqueous suspension of AgNPs as feed. The aqueous Ag-HEC nanosuspension, produced at ISTEC-CNR (Faenza (RA), Italy), at a concentration of 0.05% *w*/*w* with the addition of polyvinyl alcohol (PVA) to increase adhesion to the substrate, was sprayed on polyester textile provided by MICA NanoTech Ltd. (Limerick, Ireland). The lab-scaled automatic spray-coating machine (AUREL SPA, Modigliana (FC), Italy), located at ISTEC-CNR (Faenza (RA), Italy), has a single tunable pressure and flow rate spray nozzle. The spray chamber volume is 0.81 m^3^. The spray coasting machine is sealed in a chamber with no aspirating ventilation from the surrounding environment. Spraying was conducting under the following experimental conditions:

- deposition velocity: 1 cm s^−1^;
- deposition slab temperature: 40 °C
- coated surface: 12 cm × 12 cm
- nozzle-textile distance: 9 cm

Each spray transit lasted 1 min. After the spray, one minute was allowed to elapse before the spray chamber was opened. The experiment consisted of six tests during which the number of transits and pressure were modified. The chronological time-sheets drawn up for each test are summarized in Table 1. The background was measured before spraying was started and while the spray-coater was turned off.

### 2.2. Physicochemical Characterization of AgHEC

AgHEC NPs were characterized with a Zetasizer nano ZSP (model ZEN5600, Malvern Instruments, Malvern, UK), diluted 1:100 in water. The particle size distribution was determined by the dynamic light scattering (DLS) technique. Zeta potential measurements were performed by electrophoretic light scattering (ELS). The Smoluchowski equation was applied to convert the electrophoretic mobility to the zeta potential. DLS analysis also provides a polydispersity index (PDI), ranging from 0 to 1, quantifying the degree of colloidal dispersion; for a PDI below 0.2, a sol can be considered monodispersed. The hydrodynamic diameter and zeta potential values were obtained by averaging three measurements. Morphological analysis was performed by transmission electron microscopy (TEM). After appropriate dilution in water, one drop of AgHEC NPs was deposited on a carbon film-coated copper grid and then air dried. The sample was examined under a FEI TECNAI F20 microscope (FEI, Hillsboro, OR, USA) operating at 200 keV.

### 2.3. Methods

The measurement strategy consisted of simultaneously detecting/sampling at two locations: inside and outside (far field) the spray chamber to measure AgNP concentrations released. The far-field measurement station was positioned about 4 m from the spray machine. Monitoring included both real-time measurements and post-campaign off-line analysis of particulate matter collected on filters. Inside the spray chamber, the particle number and mass concentrations were obtained by means of a low-cost optical particle counter (OPC SPS30, Sensirion, Stäfa, Switzerland). Off-line measurements were made of airborne particle samples on polytetrafluoroethylene (PTFE) filters (1 µm porosity) and on polycarbonate (PC, 0.2 µm porosity) by means of a dual pump (Bulldog, XearPro, Cogliate (MB) Italy) at a 4 L min^−1^ flow rate for each sampling line. The FF sampling position included a diffusion size classifier (DiSCmini, Testo, Pittsburgh, PA, USA), which can determine the lung-deposited surface area (LDSA, expressed in μm^2^ cm^−^^3^) and the particle number concentration for particles in the 10–300 nm size range, as well as an aerosol photometer (DustTrack II, TSI, Shoreview, MN, USA, mod. 8520).

We used a field emission scanning electron microscope (FESEM—Carl Zeiss Sigma NTS, Gmbh Öberkochen, Jena, Germany) coupled with an energy-dispersive X-ray (EDX) micro-analyzer (EDS, mod. INCA Energy 300, Oxford Instruments, Abingdon, UK) to analyze particles collected on the filters. We recorded FESEM images at different magnifications on a piece of PC filter attached onto an aluminum holder using carbon tape. Samples were gold-coated (thickness = 5 nm). FESEM analysis allowed the assessment of overall particle distribution on the filter, and a qualitative evaluation of particle concentration. Quantitative size distribution was acquired by FESEM using ImageJ 1.52p software (National Institute of Health, Bethesda, MD, USA) for more than 200 particles.

**Figure 1:**
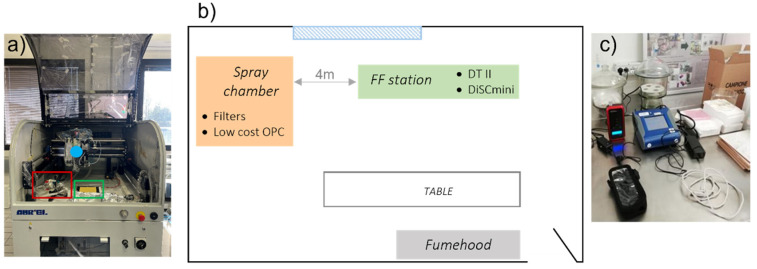
(**a**) Picture shows the spray chamber: spraying surface (green framed), spray gun (blue dot), low-cost OPC sensor, and two filter holders (red framed); (**b**) schematisation of the measurement room and instrumentation position; (**c**) picture shows the FF station at a distance of 4 m with DustTrack II and DiSCmini.

The spray chamber (Figure 1a) was equipped with two filters, one for gravimetric particulate matter (PM) assessment (polytetrafluoroethylene (PTFE) filter), one for SEM observations (polycarbonate (PC) filter), as well as an OPC SPS30. Table 2 reports the time sheet of the filters collected. Gravimetric PM on the PTFE filters was determined by a 10 µg sensitivity analytical balance. Outside the spray chamber (Far Field, Figure 1c), an aerosol photometer (DustTrack II, TSI, Shoreview, MN, USA) and a DiSCmini (Testo, Pittsburgh, PA, USA) measured the LDSA and particle number concentration.

## 3. Results and Discussion

### 3.1. Physicochemical Characterization of AgHEC

**Figure 2:**
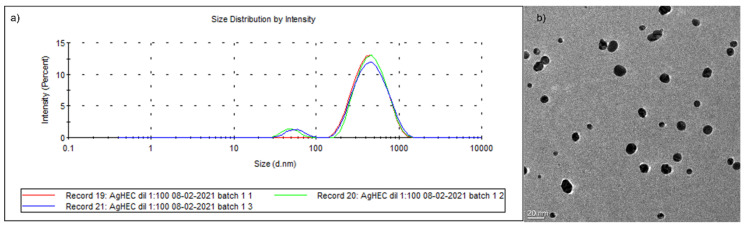
*(***a**) Particle size distribution and *(***b**) TEM image of AgHEC NPs.

AgHEC NPs were prepared according to a patented eco-friendly and easily scalable process conducted at room temperature promoting highly stable and concentrated water-based suspensions, where hydroxyethylcellulose acts both as a reducing and chelating agent. The AgHEC suspension was colloidally analyzed by DLS (Figure 2a) and ELS techniques. The suspension had a hydrodynamic diameter of 342 ± 10 nm with PDI equal to 0.4 and a zeta potential of + 14.5 ± 0.1 mV. The good correlation between curves of three measurements (Figure 2a), the very low standard deviation and relatively low PDI demonstrated the presence of a colloidally stable and monodispersed suspension. The zeta potential value observed corresponds to the positive surface charge of Ag NPs surrounded by the HEC coating. The TEM image (Figure 2b) shows well-dispersed spherical particles with primary diameter ranges from 3 to 20 nm, lower than the DLS hydrodynamic diameter, confirming the presence of HEC coating on AgNPs. This and an almost total reaction yield were further demonstrated by FTIR and XRD analysis, respectively, as reported in our previous paper [14].

### 3.2. Data Analysis (Inside Chamber)

**Figure 3:**
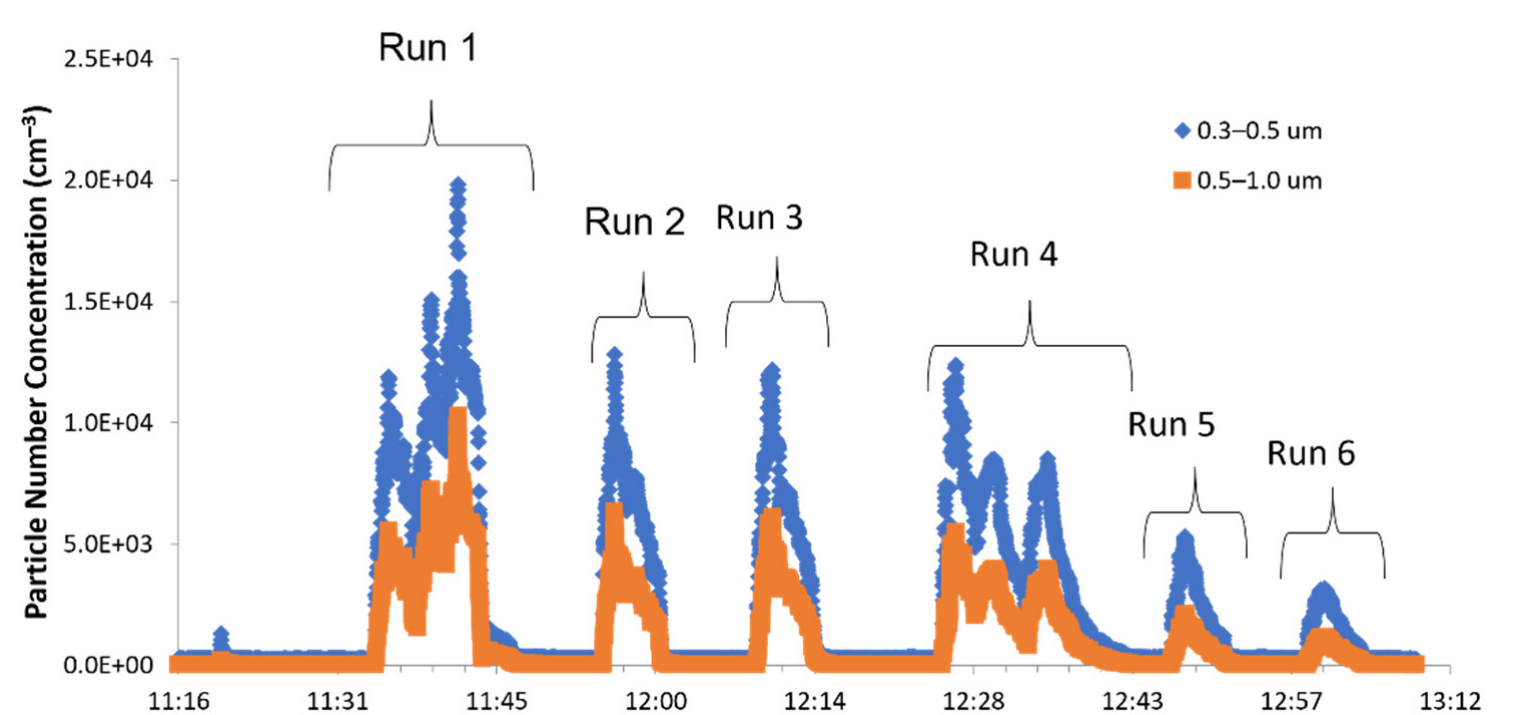
Temporal trend of the particle number concentrations inside the spray chamber.

**Figure 4:**
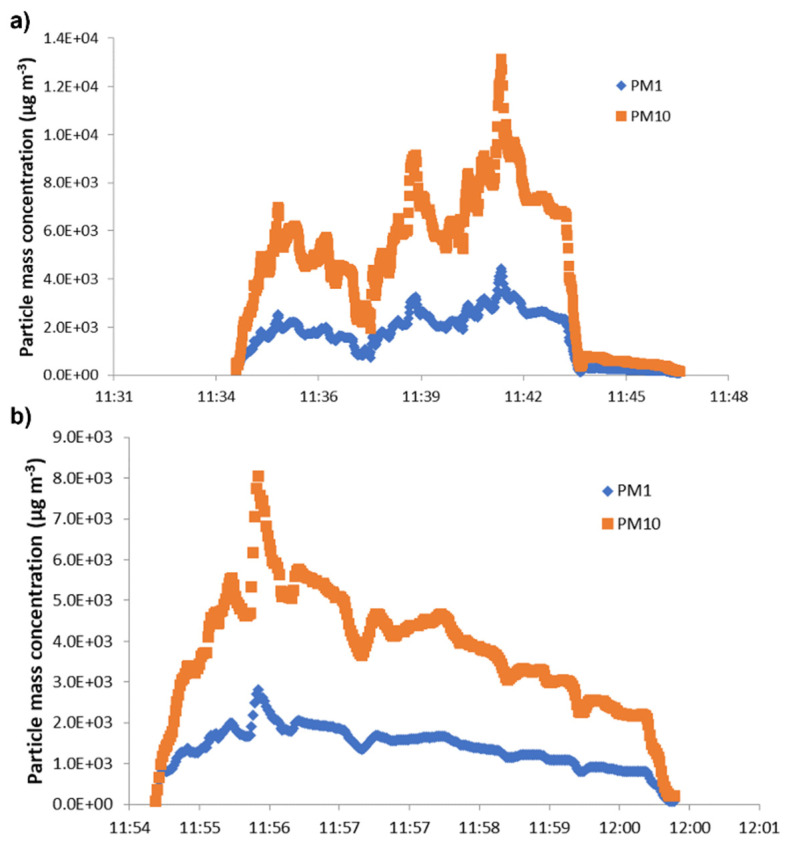
PM1 and PM10 particle mass concentration measured within the spray chamber for (**a**) run 1 (three spray transits) and (**b**) run 2 (one spray transit). Both tests were performed at 1 bar nozzle pressure. The temporal distance between spray coatings (run 1) was about 2/3 min.

Particle numbers and mass concentrations were determined for all six spray tests. Figure 3 shows particle number concentrations in the spray chamber in the size ranges 0.3–0.5 µm and 0.5–1.0 µm observed with the OPC SPS30. The test performed at lower pressure (1 bar), corresponding to run 1–3, showed higher particle number concentrations than the test at a higher pressure (1.5 bar). Test runs 4 to 6, however, showed lower particle number concentrations. Higher fine particle concentrations (0.3–0.5 µm) compared to coarser particles were found for all measurements (Figure 3). The three spray transits of tests 1 and 4 are shown by the three spikes in the particle number concentration pattern. This clearly indicates the particle number concentration during spraying activity. The differences between one and three transits are more clearly shown in Figure 4, where PM1 and PM10 particle mass concentrations measured by means of the SPS30 OPC inside the spray chamber are reported for run 1 (Figure 4a) and run 2 (Figure 4b). The temporal trend (Figure 3) shows a good correspondence between spray activities and particle number concentration increase, with a consequent sudden (runs 1–3 at 1 bar) and gradual (runs 4–6 at 1.5 bar) fall off after spray-process cessation until background-level restoration.

**Figure 5:**
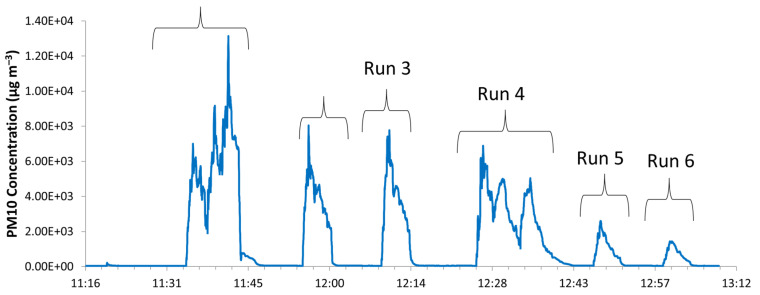
Temporal trend of PM10 concentrations by means of OPC SPS30.

**Figure 6:**
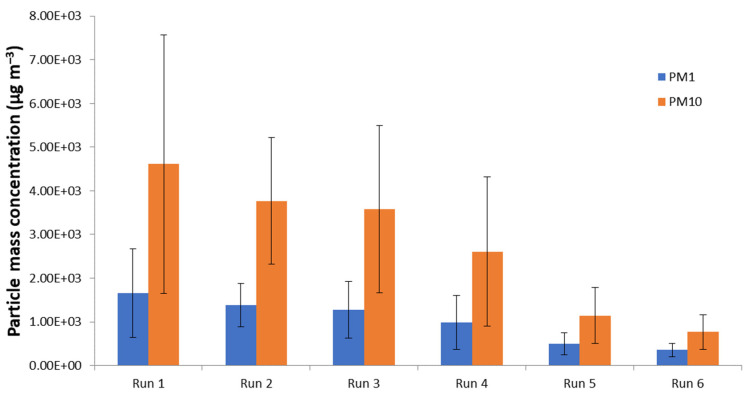
Averaged PM1 and PM10 particle mass concentrations for each test. Bars show particle mass concentration variability. Data collected by OPC SPS30 sensor.

PM10 concentrations are showed in Figure 5. Comparison between different working nozzle pressures showed higher particle mass concentrations at 1 bar than at 1.5 bar (Figure 6), as observed also by the particle number concentration data reported in Figure 3. This was further confirmed by gravimetric particulate matter determination on the filters collected: 222 µg m^−3^ at 1 bar (run 1–3) and 114 µg m^−3^ at 1.5 bar (run 4–6).

**Figure 7:**
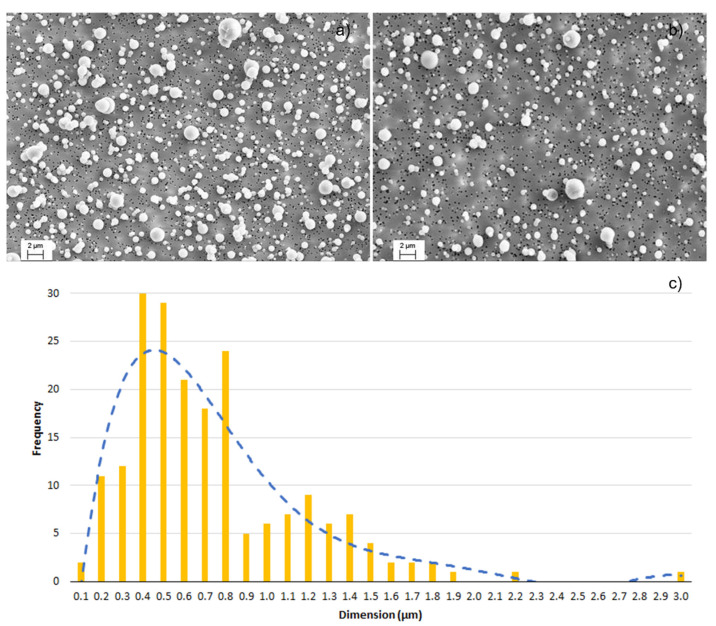
SEM images of *(***a**) filter 1 (run 2 at 1 bar); *(***b**) filter 2 (run 5 and 6 at 1.5 bar); *(***c**) size distribution of AgHEC particles after spray-coating activities (mode: 0.39 µm), from SEM images.

**Figure 8:**
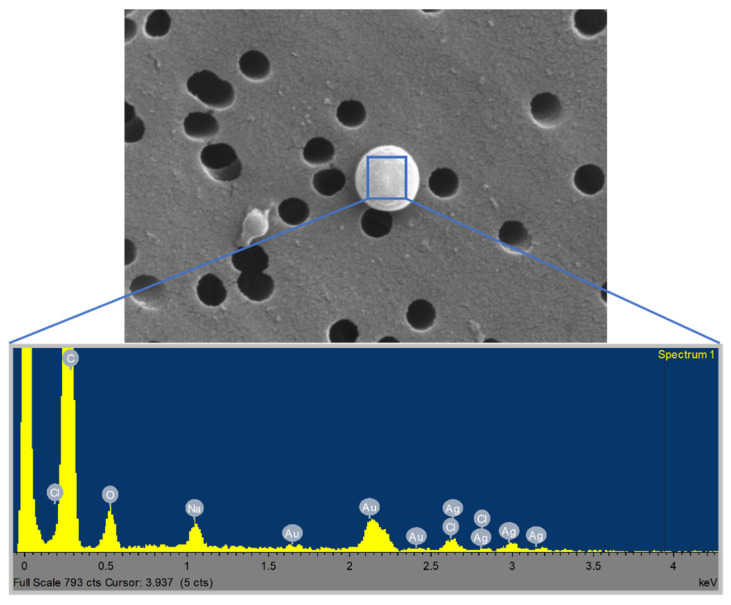
SEM-EDX analysis on particle deposited on filter.

The nozzle pressure is related to the amount of air atomized, which in turn signifies the suspension dilution. A higher nozzle pressure therefore corresponds to higher dilution rates and lower particle concentrations [16]. Further qualitative validation was provided by SEM images of the filters (Figure 7). At 1 bar (Figure 7a), a high quantity of spherical particles was observed. Inversely, fewer particles were found on filters collected during runs at 1.5 bar (Figure 7b). In addition, the higher the nozzle pressure, the finer the atomized droplets and the faster the water evaporation [17]. In fact, as reported in the literature [18], the working pressure is the main parameter affecting the droplet size distribution in spraying processes. Moreover, SEM analysis detects micrometric and sub-micrometric granules with a widely differing particle size distribution (Figure 7c–average value 0.74 ± 0.43µm). These granules consisted of primary Ag-HEC characterized by typical NP diameters ranging from 3 to 20 nm and formed during the spray process that leads to NPs agglomeration. SEM-EDX analysis (Figure 8) confirms the presence of Ag NPs on granules atomised during spraying. In fact, the spectrum reported in Figure 8 shows the presence of Ag as well as Na and Cl due to by-product synthesis, as explained in Table 3. Au was also detected during SEM sample preparation.

### 3.3. Process Optimisation

The data collected were used for process optimization by calculating deposition efficiency, as follows:

(1) $$E=\frac{Q_{\mathrm{out}}}{Q_{\mathrm{in}}}$$

where Q_in_ is the atomized suspension and Q_out_ is the deposited material.

**Figure 9:**
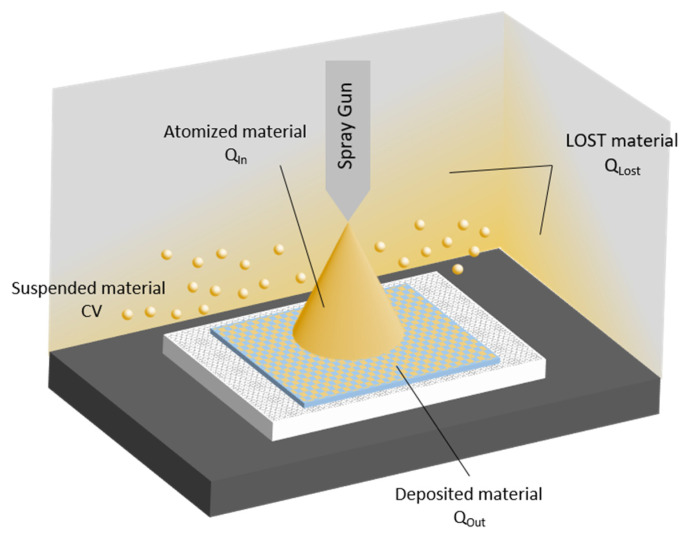
Scheme of the mass balance inside the spray chamber.

The material not deposited on the substrate may either be deposited on other surfaces (Q_lost_) or suspended inside the chamber (C × V). Taking all terms into account, the equation representing the mass balance schematized in Figure 9 is as follows:

Q_in_ = Q_out_ + Q_lost_ + C × V (2)

where C is the airborne concentration measured inside the chamber at the end of the spray (by means of the OPC SPS30 sensor, Sensirion, Stäfa, Switzerland), and V is the chamber volume. Q_out_ is obtained by weighing textile samples before and after spraying and water evaporation.

The composition of the Ag-based coating is reported in Table 3. Table 4 shows the breakdown of the sprayed material actually deposited on the textile support and material lost. The difference between Q_in_ and Q_out_ is the amount of material deposited on surfaces other than the textile pieces, and the material remaining airborne, which was evaluated by measuring particle concentration in the spray chamber. It was observed that an increase in spraying pressure decreased the amount of material deposited. In addition, multiple sprays (runs 1 and 4) led to an increase in material lost. The deposition efficiency (E) and hence process performance were calculated using Equations (1) and (2). The results are reported in Table 4. We observed that increasing the working pressure from 1 to 1.5 bar increased the deposition efficiency. On the other hand, lower efficiency rates were found for multiple sprays (run 1 and 4) at the same nozzle pressure compared to single transit tests.

### 3.4. Data Analysis (Outside Chamber)

**Figure 10:**
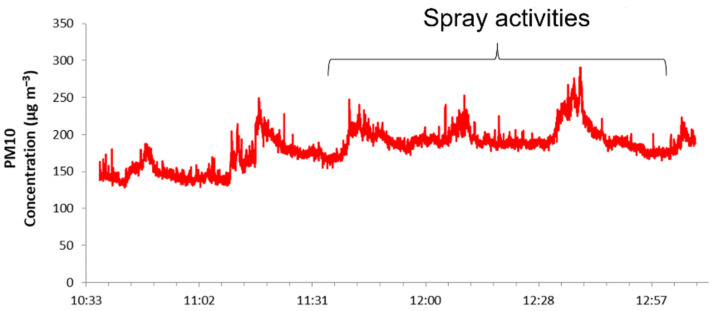
Temporal trend of particle mass concentrations, in terms of PM10, measured by means of DustTrack II at far field.

The measurements performed outside the spray chamber (Figure 1c) determined the silver particles released into the environment by the spray-coating process. The aerosol photometer located outside the spray chamber in a far-field position showed only a small increase in particle mass concentration during the sprays, as shown by the PM10 concentration trend in Figure 10. After each run, the window of the spray chamber was opened to set up the next test and so a certain amount of aerosol escaped into the room. The spikes in particle mass concentration measured at far field (Figure 10) a short time after the end of spraying may correspond to the moment when the spray chamber window was opened, releasing airborne material. It may, however, be assumed that during spraying, there was negligible release of aerosolized particles from the machine into the environment.

**Figure 11:**
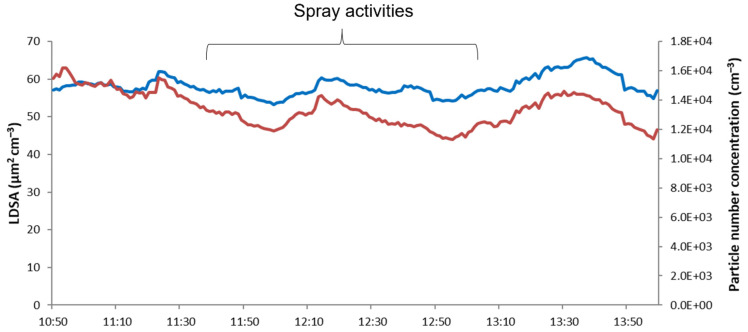
Temporal trend of LDSA (blue line) and particle number concentrations (red line) measured by DiSCmini.

Further confirmation was given by the DiSCmini findings (in the far field) for the lung-deposited surface area (LDSA) and particle number concentration (Figure 11). LDSA is a metric that takes into account the deposition efficiency of airborne objects in different compartments of the lung [19,20,21]. LDSA data for occupational activities involving engineered ultra-fine particles (UFP) are scant, and often concern occupational scenarios such as welding activities where values can be as much as 137 µm^2^ cm^−^^3^. In different urban areas, average LDSA values are usually below 100 µm^2^ cm^−^^3^ [22], evidencing the important contribution of indoor activities as sources of ultrafine particle (UFP) exposure. In our experimental conditions, LDSA values were found to be lower than 70 µm^2^ cm^−^^3^, much lower than the peak value emitted by a candle burning (about 250 µm^2^ cm^−^^3^) [23], thus demonstrating the negligible contribution of these spray processes as a critical source of particle inhalation exposure. Neither graphic trend shows any increasing value due to emissions from the spray machine (Figure 11).

**Figure 12:**
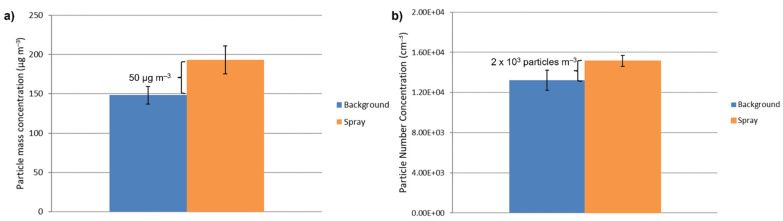
(**a**) Particle mass concentrations measured by means of the DustTrack and (**b**) particle number concentrations measured by means of the DiSCmini. Averages concentration before starting the coatings (Background) and at the end of the process (Spray). Bars show the variability of the concentration (one standard deviation).

Following an approach proposed by ISO in technical specification ISO/TS 12901-2:2014 [24], the values observed were compared to the background concentrations. Figure 12 shows the concentrations detected before starting (background) and at the end of the spraying process (spray). We compared the concentration levels as measured by the DustTrack (particle mass concentration) and the DiSCmini (particle number concentration), calculating the exposure concentrations as the difference between measured and background concentrations. Compliance with occupational limits was assessed by comparing our data with the occupational exposure limit (OEL) reported by NIOSH [25]. Considering an OEL for inhalable silver of 100 µg m^−^^3^, we observed that the particle mass concentration (50 µg m^−^^3^) associated with the spray process used (Figure 12a) stayed below the OEL, leading to the conclusion that the spray coating process employed in our experimental condition does not represent a critical scenario in terms of worker exposure. The values recorded during spraying allow evaluation of the incidence of a particular task or event on background level concentration and of workplace environmental quality [26]. Seipenbusch and colleagues [27] suggest that the average background concentration of indoor nanoaerosols should be in the range of 1 × 10^3^ to 10 × 10^3^ particles cm^−^^3^. With an averaged particle number concentration of around 13 × 10^3^ particles cm^−^^3^ (Figure 12b), our indoor environment cannot be considered completely clean. However, the increase of not more than 2 × 10^3^ particles cm^−^^3^ compared to background (Figure 12b) remains below the safety limit, expressed as nano referenced values (NRVs). In fact, the threshold concentration proposed for the introduction of appropriate exposure control measures in the case of particles with a density higher than 6 g cm^−^^3^, such as Ag, is 20 × 10^3^ particles cm^−^^3^, expressed as an NRV [26]. These important findings suggest that the particle exposure connected with spray coating stays below the limits set, both in terms of the particle mass and number concentration. The total enclosure of the spray system in a chamber, as described in Section 2.1, is the main factor contributing to these positive results. In fact, a more critical scenario was found in a spray system described in our previous study [10] that was not sealed off from the environmental. Nonetheless, an important addition to our spray process will be the introduction of a ventilation system connected to the spray chamber.

## 4. Conclusions

Antibacterial textiles were produced applying Ag-HEC nanoparticles by a spray-deposition technique using a lab-scaled automatic spray-coating machine. Measuring particle emissions inside and outside the spray-coating chamber allowed us to identify the experimental conditions under which material deposition was maximized, thereby minimizing the amount of material lost, as well as to estimate AgNP exposure during spray operations. Both aspects are relevant for the definition of safe and sustainable-by-process-design strategies to control and reduce the risk of nano-manufacturing processes. More specifically, the monitoring campaign allowed us to determine deposited, lost, and airborne material and so calculate the deposition efficiency. The results showed that increasing the spray pressure from 1 to 1.5 bars increases the E, while increasing the number of sprays lowers the E, demonstrating that the multiple spray system was less efficient than single spray method. The AgNP exposure observed suggests that under our experimental conditions the spray-coating process does not represent a critical scenario in terms of worker exposure. It should, however, be underlined that the sealed-chamber design of the spray system used was a key factor in keeping down airborne particle concentration.

To the best of our knowledge, this is the first study in the literature that has used in-process performance data both for deposition optimization purposes and to evaluate particle exposure concentrations.

## Figures and Tables

**Table 1:** Description and time sheet of measurements performed on spray-coating machine.

Run	Number of Transits	Pressure (Bar)	Starting/Ending Time
1	3	1	11:35–11:42
2	1	1	11:56–11:57
3	1	1	12:10–12:11
4	3	1.5	12:27–12:35
5	1	1.5	12:47–12:48
6	1	1.5	13:00–13:01

**Table 2:** Time-sheet of filters collected inside the spray chamber.

Test	Filter Gravimetric PM	Filter SEM
1	Filter 1	
2		Filter SEM 1
3		
4	Filter 2	
5		Filter SEM 2

**Table 3:** Composition (% *w*/*w*) of aqueous Ag-based coating sprayed on polyester textile substrate.

Ag	HEC	NaCl *	PVA **
0.5	0.455	0.0755	0.5

* synthesis by-product, formed by Cl^−^ (conter ion of hydroxyethylcellulose compound) and Na^+^ (in NaOH added as catalyst of reduction rection of AgNO_3_, precursor of AgNPs). ** added to improve coating adhesion on polyester textile substrate.

**Table 4:** Atomised suspension, deposited and lost materials, aerosol concentration and deposition efficiency (E) for each run.

Run	Ag-HEC Suspension Consumption (g)	Q_in_ * (mg)	Q_out_ (mg)	Airborne Material ** (mg)	Q_lost_ (mg)	E
1	23.0	248.4	54.4	9.0	185.0	0.22
2	8.4	90.9	22.9	7.5	60.6	0.25
3	12.0	129.3	25.4	8.0	95.8	0.20
4	16.4	177.6	54.7	6.9	116.0	0.31
5	3.0	32.0	13.2	2.9	15.9	0.41
6	1.8	19.4	8.3	2.4	8.7	0.43

* Atomized suspension was obtained by considering percentages of each component (Ag, HEC, NaCl and PVA, see Table 3). ** Airborne material was computed by considering the measured concentration inside the spray chamber times the chamber volume (CV, as indicated in Equation (2)).
